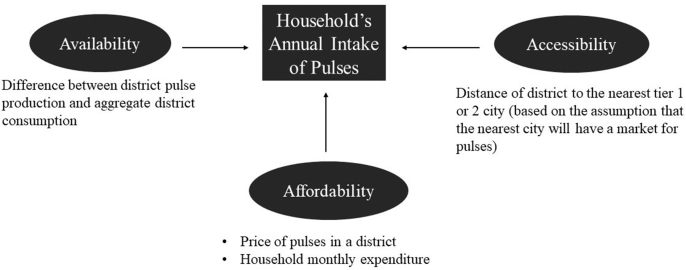# Factors influencing household pulse consumption in India: A multilevel model analysis

**DOI:** 10.1016/j.gfs.2021.100534

**Published:** 2021-06

**Authors:** Anjaly Teresa John, Sanchit Makkar, Sumathi Swaminathan, Sumedha Minocha, Patrick Webb, Anura V. Kurpad, Tinku Thomas

**Affiliations:** a St. John's Research Institute, Bangalore, India; b Friedman School of Nutrition Science and Policy, Tufts University, Boston, USA; c St. John's Medical College, Bangalore, India

**Keywords:** Pulses, India, Availability, Accessibility, Affordability, Multilevel model, APS, Area and Production Statistics, CES, Consumer Expenditure Survey, CI, Confidence Interval, g, Grams, HH, Household, kg, Kilograms, MLM, Multilevel Model, NSSO, National Sample Survey Office, p.a., Per annum, p.c., Per capita, PDS, Public Distribution System, SC, Scheduled Caste, ST, Scheduled Tribe, VDSA, Village Dynamics in South Asia, VPC, Variance Partition Coefficient

## Abstract

Pulses (also known as legumes) are important in achieving nutrient adequacy in India due to their quality protein content. This study compared district-level pulse production and consumption across India, and household and district-level determinants of pulse intake, including availability, accessibility and affordability, using multi-level models in nationally representative datasets for 2011–12. The per capita consumption was about 50% of recommended intake (80 g/day), even in high-producing districts. District-level pulse production was associated with household pulse intake (2.73 × 10^−8^ [5.19 × 10^−9^, 4.94 × 10^−8^]) and market accessibility (−0.0077 [-0.0133, −0.0021]). Affordability (absolute price of pulse) was also associated with household intake. While agricultural policies relating to pulses have been oriented towards improving pulse output and productivity, forward-looking policies to improve pulse intake should focus on demand-side factors, such as improved market accessibility and the affordability of pulses relative to other foods.

## Highlights

• Pulse production exceeded the recommended intake of their population but consumption met only 50% of recommended intake.
• Effects of pulses availability, accessibility and affordability on pulse consumption were explored by multilevel model.
• District-level availability, affordability and accessibility were significantly associated with pulse intake.
• Household pulse production is associated with pulse intake; advocating production in rural areas can improve consumption.

## 1 Introduction

Pulses and legumes have been produced and consumed in India over millennia (Nene, 2006), but the contribution of pulse production to total food grain production has declined from 16.6% in 1950–51 to 7% in 2014–15, partly due to the stagnation in pulse production technology and acreage under pulse cultivation since the Green Revolution (Tiwari and Shivhare, 2016). Growth in pulse production has also trailed far behind that of other food crops, particularly cereals, which saw an increase in production to the tune of 280% in the last six decades, compared with only 32% growth for pulses (Vilas et al., 2018). Pulse availability too declined from 60 g per capita per day in 1951 to 47 g in 2014 (Tiwari and Shivhare, 2016). Pulse consumption reduced from 0.81 kg to 0.96 kg per capita per month in rural and urban India respectively in 1999–2000 to 0.74 kg and 0.86 kg in 2011–12 (National Sample Survey Office, 2001, 2014a). Although a direct link between the fall in pulse production and consumption has not been established, there seems to be an association between the two.

Currently, India is the largest producer and consumer of pulses in the world, but consumption exceeds production and is therefore supported by imports. In 2014–15, India produced 17.4 million tonnes of pulses and consumed 22.7 million tonnes, with imports amounting to 4.6 million tonnes (Umanath et al., 2016). The gap between demand and domestic supply of pulses is expected to widen over time. Vilas et al. (2018) project the demand-supply gap for chickpea and pigeon pea widening from 4.8 million tonnes in 2025 to 11.5 million tonnes in 2030. Minocha et al. (2019) projected the economic demand for pulses to be about 26 million tonnes by 2026, and the availability to fall short by 2 million tonnes. Pulse production will have to grow at an annual rate of 2.2% to meet projected demand of 39 million tonnes in 2050 (Indian Institute of Pulse Research, 2015), although pulse production has been volatile in the period 2012–2017 with growth fluctuating between −11% in 2014–15 and + 40% in 2016–17 (Directorate of Pulses Development, 2017).

In the early 2010s, cereals contributed about 58% and 49% of total protein intake in rural and urban India, respectively (National Sample Survey Office, 2014b), but pulse protein has higher amounts of indispensable amino acids, such as lysine, compared to cereal protein which is an indicator of the protein quality. Thus, pulses enjoy a special position in the Indian diet because they have the potential to reduce quality protein deficiency, when animal source foods are limited because of affordability and perishability as well as for cultural reasons. As a result, India's National Institute of Nutrition (2011) recommends a daily pulse intake of 80 g per person/day. The risk of quality protein deficiency (based on Estimated Average Requirements for each age group) ranges from 4% to 26% for different age groups and sectors in India (Minocha et al., 2019). By 2026, projected availability would meet about 57– 87% of pulse intake required to minimize the risk of dietary quality protein inadequacy to a very low level (Minocha et al., 2019). But this is not true for the whole of India because there is variability in production and consumption across the states as well as districts. It is important to understand this variability and the drivers of variability in consumption across India. Although pulses are the cheapest source of non-cereal plant protein in India (Joshi et al., 2016), their price is highly variable. The volatility of pulse prices has a cyclical nature on pulse production and a ‘cobweb phenomenon’ whereby prices have a lagged effect on production. Farmers base their production decisions on prices observed in the previous period, which results in an under or over-production of pulses, causing price cyclicality (Joshi et al., 2017).

The role of market accessibility has not been adequately explored in relation to pulse consumption in India. Sibhatu et al. (2015), Luckett et al. (2015) and Koppmair et al. (2016) have indicated that better market accessibility is associated with higher dietary diversity, but its role in pulse inclusion in the diet is poorly understood. Socio-economic and demographic factors are also important determinants of a household's food consumption. Mfikwa and Kilima (2014) indicated that, among rural households, the decision to consume pulses was influenced by household size and educational attainment of the household head while among the urban households the extent of consumption was additionally influenced by the household's non-food expenditure, and by the relative prices of pulses and meat.

The present study examines the distribution of consumption and production of pulses across districts of India to identify drivers of household consumption of pulses, particularly with respect to household and district-level factors of availability, accessibility and affordability.

## 2 Methods

The pulses considered in this study were pigeon pea (arhar/tur), gram/chickpea, green gram (moong), red lentils (masoor), black gram (urad), dried peas and grass pea (khesari). Production statistics for other pulses and legumes such as cowpea (lobia), moth, guar seed, horsegram, lentils, other kharif and rabi pulses are also available for districts of India. However, these were not included in the analysis as data on consumption was not available for these items in the Consumer Expenditure Survey. We do not consider soya as a pulse in this study, because its production is mainly used towards processed vegetable oil, livestock feed and industrial applications such as fatty acids, soaps and biodiesel (Hazra et al., 2016). Products derived from pulses such as pulse flour (*besan* and *sattu)* were also included in household consumption. However, pulses consumed as a part of meals consumed outside the house or packaged foods were not considered in the analysis.

### 2.1 Sources of data

The data from the National Sample Survey Office's 68th Round of the Consumer Expenditure Survey (CES, 2011–12) (Type 1) (National Sample Survey Office, 2013) and district-wise annual production of pulses (2011–12) from Area and Production Statistics (APS), Ministry of Agriculture and Farmers' Welfare (Directorate of Economics and Statistics & Ministry of Agriculture and Farmers' Welfare) were used for this study. We did not use NSSO CES Type 2, as the data on consumption of certain food groups were collected using a 7-day reference period, whereas Type 1 data collection was based on a 30-day reference period for food items, including 30 and 365-days reference periods for some non-food items. District data were available for 623 out of 640 districts of India as of 2012 and all analyses, including the household analyses, were restricted to these districts.

### 2.2 Data considered for analysis

The ninth quinquennial Household Consumer Expenditure survey (CES) of the 68th round of the NSSO covered all regions of India (29 states and 6 union territories, across 7469 villages and 5268 urban blocks), except a few interior villages. About 69% of the surveyed households among the 623 districts included in the analysis belonged to rural areas. Households were selected by multi-stage stratified sampling. Monthly per capita consumer expenditure as well as the household food purchase of 223 food items were collected through this survey, for a recall period of 30 days. The total quantity of pulses and pulse products purchased per household per month was computed, and this proxied for monthly household pulse consumption (kg). Data on socio-demographic characteristics of the household and total monthly per capita expenditure were also used. About 101,000 households were considered for the analysis.

The Food and Agriculture Organization (2008) notes that food availability at a national level is determined by level of domestic food production, loss and waste, stock levels and net trade, but due to unavailability of data on the latter two, we used district production surplus (after accounting for wastage) as an indicator of availability which is recognised as a determinant of local availability (Pangaribowo et al., 2013). The local availability was the surplus of the district cumulative production of pulses computed with data available from Area Production Statistics (APS) for the year 2011–12, over the aggregate district pulse consumption computed with data available from NSSO 2011–12. District production surplus was measured as the difference between aggregate district pulse production and aggregate district consumption. District per capita intake was multiplied by district population from Census (2011) to obtain aggregate district consumption.

The availability of pulses was also examined as surplus of production in a district over the aggregate recommended intake for the district. For this, the annual per capita recommended pulse intake (based on a per capita recommended intake of 80 g/day for a moderately active person (National Institute of Nutrition, 2011) was multiplied by district population to arrive at the aggregate recommended pulse intake for the district, and this was subtracted from the district pulse production to compute district production surplus over the aggregate recommended intake for the district. The availability figures were adjusted for production loss of 22.5% by subtracting this quantity from the district production (Post-Harvest Systems of Pulses in India). Local availability of pulses, as surplus of district pulse production in excess of district aggregate consumption, was a district-level covariate.

Intakes were compared across production-surplus and deficit districts, and across high and low-producing districts. Production-surplus districts were those where the district availability (i.e. difference between district aggregate production and consumption) was positive. For the latter comparison, districts were classified as ‘high-producing’ if the per capita annual production was greater than the national per capita annual production (9.51 kg) and as ‘low-producing’ otherwise.

The *accessibility* of pulses was assessed as the accessibility to pulse market and distance to market is considered a good indicator of accessibility (Kruseman et al., 2006). Due to the dearth of data in NSSO CES on actual markets accessed by the district both by household and retailers, we assumed that the nearest tier I or tier II city to the district (which could lie outside the state too) would have a functioning market for pulse purchase. Therefore, the distance of the district to the nearest tier 1 or 2 city was used as an indicator of accessibility. The distance to 96 tier I and II cities across the country were considered for the analysis. The distance to market was a district level covariate.

Pulse *affordability* depends on household total income and the price of the food group (pulses), after controlling for price of other food items. Income affordability was proxied using households' monthly per capita expenditure (MPCE) given in the CES. The MPCE was multiplied into the household size to arrive at the household's total monthly expenditure. The price of pulses for the district for year 2011–12 was the district-level median price of each pulse in the CES. To obtain weighted price, the price of each type of pulse was weighted by its percent contribution to the overall pulse consumption in the district. The details of computation are presented in Supplementary Material 1. Thus, weighted pulse price was a district level variable. Similarly, a single price index for all other food items was calculated for each district. For this, district level median price of each food item was weighted with its percent contribution to overall food consumption (excluding pulses) in the district, and the resulting figures were summed for all food items. The study additionally explored the association of income status of households that reported pulse consumption from own production with annual household pulse intake. Households were classified into expenditure quintiles based on the monthly per capita expenditure from CES.

The economic strength of the district was controlled for by using the median monthly per capita expenditure of the district. The household-level characteristics considered in the model were household size, total land owned (hectares), maximum educational attainment by a woman (in years), whether the household belongs to the general category, religion (explored separately), whether the household is a beneficiary of the Public Distribution System (PDS). The PDS was considered because a few states provide pulses at a subsidized rate to the beneficiary household (Agarwal, 2020).

### 2.3 Statistical methods

The surplus pulse production of the district in comparison to aggregate district consumption and recommended consumption are presented separately in two choropleth maps to represent the variations or patterns in pulse production surplus across geographical regions (The Data Visualization Catalogue - Choropleth Map). Comparisons of pulse consumption between high and low producing districts (based on a cut-off of national per capita production of 9.51 kg), as well as between production-surplus and deficit districts and between households that consumed pulses from own production and those that did not, were done using Mann-Whitney *U* test.

The framework for analysing household intake of pulses is presented in Supplemental Figure 1. When there is evidence that data is grouped or clustered, an Ordinary Least Squares (OLS) regression violates the Gauss Markov assumption that the observations are independent of each other and that the residuals are uncorrelated with each other. While we can run a fixed effects model by using a unique identifier for districts, since the number is large (623 districts), the parametrization might lead to unreliable estimates. In addition, OLS regression ignoring the district clustering can underestimate the standard errors, leading to narrower confidence intervals and smaller p-values. A multi-level model approach was adopted to deal with the hierarchical, clustered structure of the data, as well as to understand the contextual effects of availability, affordability and accessibility on a household's intake of pulses along with household characteristics. The model specification is given as

$$Y_{ij}=\beta_{0}+\beta_{1}X_{1ij}+\beta_{2}X_{2j}+\zeta_{j}$$

Where $Y_{ij}$ is the response variable (monthly household pulse consumption) for household i in jth district, $X_{1ij}$ is the household level (Level 1) covariates for household i in district j, and $X_{2j}$ is the district level covariates (Level 2) for all households in district j, $\zeta_{j}\sim N\left(0,\psi \right)$ a district specific random intercept. Regression coefficient with 95% confidence interval are reported. The variance partition coefficient (VPC) in the null model (no covariates) is reported. Additionally, we explored the association of these covariates with pulse intake by adult males in the household, using consumer units (Gopalan et al., 1989).

The aggregation analysis and mapping of districts to nearest cities was carried out on R version 3.4.2 and the multilevel modelling was done using Stata 16 (StataCorp. 2019. STATA Statistical Software: Release 16. College Station, TX: StataCorp LLC.).

## 3 Results

### 3.1 Consumption and production of pulses across districts

**Fig. 1:**
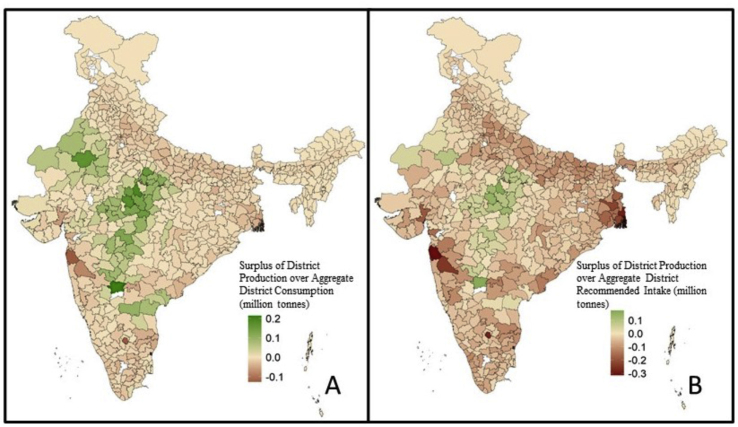
A: Surplus pulse production over consumption by districts of India in 2011–12 (in million tonnes ). B: Surplus pulse production over recommended pulse consumption of 80 g/day by districts of India in 2011–12 (National Institute of Nutrition, 2011) in million tonnes. Pulse production figures adjusted for 22.5% production loss (Post-Harvest Systems of Pulses in India). Districts for which no data was available were shaded in white. Negative surplus to be considered as deficit. Source: Authors' calculations from Crop Production Statistics Information System (Directorate of Economics and Statistics, Ministry of Agriculture and Farmers' welfare) for 2011–12 and NSSO 68th Round Consumer Expenditure Survey (2011–12).

**Fig. 2:**
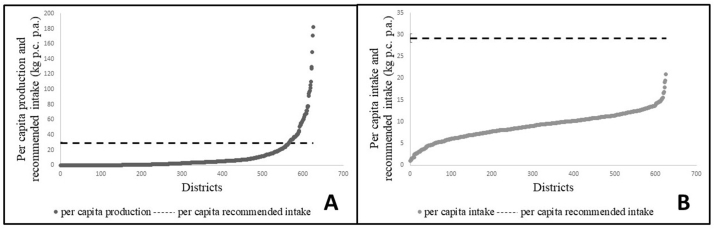
A – Pulse production across districts in India in 2011–12; Y axis: Per capita production per annum (kg); X axis: districts of India ordered in increasing order of per capita production; Dark circles represents per capita production per annum (kg); Dashed line represents recommended pulse consumption per annum (29.2 kg) B – Pulse consumption across districts in India in 2011–12; Y axis: Per capita consumption per annum (kg); X axis: districts of India ordered in increasing order of per capita consumption; Dark circles represents per capita consumption per annum (kg); Dashed line represents recommended pulse consumption per annum (29.2 kg). Source: Crop Production Statistics Information System (Directorate of Economics and Statistics, Ministry of Agriculture and Farmers' welfare) for 2011–12 and NSSO 68th Round Consumer Expenditure Survey (2011–12). Recommended pulse consumption of 80 g/day is 29.2 kg/year (National Institute of Nutrition, 2011).

**Fig. 3:**
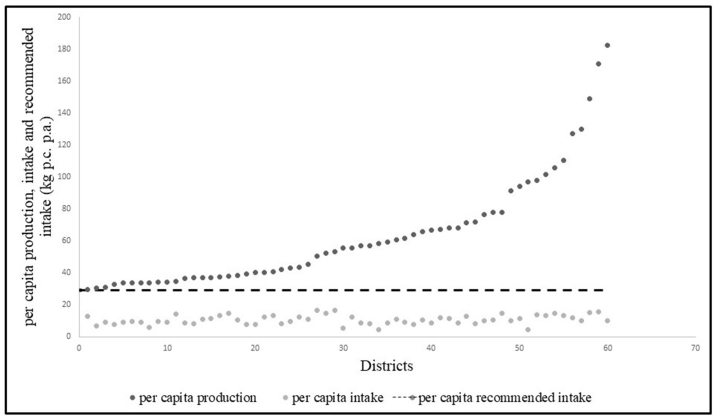
– Pulse consumption and production versus the Indian National Institute of Nutrition's recommended intake (kg per capita per annum) across 60 production-surplus districts in India (2011–12) (National Institute of Nutrition, 2011); Y axis: Per capita consumption, production and recommended intake per annum (kg); X axis: pulse surplus districts of India ordered in increasing order of per capita production; Dark circles represents per capita production per annum (kg); Empty circles represent per capita consumption; Dashed line represents recommended pulse consumption per annum (29.2 kg). Source: Crop Production Statistics Information System (Directorate of Economics and Statistics, Ministry of Agriculture and Farmers' welfare) for 2011–12 and NSSO 68th Round Consumer Expenditure Survey (2011–12). Recommended pulse consumption of 80 g/day is 29.2 kg/year (National Institute of Nutrition, 2011).

The seven pulses included in this study constituted 83.2% of all pulses produced (excluding soyabean) and 94% of all pulse and pulse products consumed in 2011–12. Annual per capita consumption slightly exceeded production (10.26 kg and 9.51 kg, respectively in Table 1). While chickpea (47.1%), followed by pigeon pea (16.5%), black gram (11.5%) and green gram (11.1%) were the most widely produced pulses, pigeon pea (30.9%) was the most widely consumed pulse followed by chickpea (23.8%), red lentils (13.9%), green gram (12.9%) and black gram (11.3%) (Supplemental Table 1). Grass pea was the least produced (2.5%) and consumed (1.4%) pulse (Supplemental Table 1). Although per capita consumption outweighed per capita production in India in 2011–12, there were 155 districts where the district pulse production was in surplus of the aggregate district consumption (Table 2, Fig. 1A). Median annual per capita consumption in these surplus districts was slightly higher than in other districts (9.7 kg vs 9 kg respectively, p = 0.02 using Mann Whitney *U* test). If a higher recommended pulse intake for the district were to be considered at 80 g/day (National Institute of Nutrition, 2011), there were 60 districts which produced sufficient quantities of the seven pulses combined (Table 2, Fig. 1B). Most of these districts (23) were in Madhya Pradesh, followed by Maharashtra and Rajasthan at 11 and 10 each. Interestingly, these states also had a high number of production deficit districts (27, 23 and 22 respectively). However, despite the sufficient production in 60 districts as indicated in Fig. 2A, all districts including the pulse production-surplus districts had per capita pulse intakes that were lower than the recommended intake (Fig. 2B), with most districts reporting per capita intake less than 50% (Fig. 3) of the recommended per capita consumption of 80 g/day (National Institute of Nutrition, 2011). The production-consumption matrix in Supplemental Table 2 indicates that in 51% of the 623 districts, per capita production and consumption were lower than median per-capita production and consumption at all-India levels.

**Table 1:** All India Intake and Production of Pulses (and Pulse Products), including pigeon pea, chickpea, green gram, red lentils, black gram, dried peas, and grass pea.

Per capita production after adjusting for production loss	9.51 kg pc pa
Per capita consumption	10.26 kg pc pa
Aggregate production adjusted for production losses	11.5 million tonnes
Aggregate Consumption	11.38 million tonnes

Source: Authors' calculations from Crop Production Statistics Information System (Directorate of Economics and Statistics, Ministry of Agriculture and Farmers’ welfare) for 2011–12 and NSSO 68th Round Consumer Expenditure Survey (2011–12).

**Table 2:** Production Surplus/Deficit Districts.

	No. of districts	Surplus/deficit (adjusted for production loss) (million tonnes per year)
Difference between aggregate district production (adjusted for production loss) and aggregate district consumption > 0	155	6.10
Difference between aggregate district production (adjusted for production loss) and aggregate district consumption < 0	468	−6.25
Sub-total	623	−0.15
Difference between aggregate district production (adjusted for production loss) and recommended intake aggregated for the district > 0	60	2.93
Difference between aggregate district production (adjusted for production loss) and recommended intake aggregated for the district < 0	563	−26.65
Sub-total	623	−23.72

Source: Authors' calculations from Crop Production Statistics Information System (Directorate of Economics and Statistics, Ministry of Agriculture and Farmers’ welfare) for 2011–12 and NSSO 68th Round Consumer Expenditure Survey (2011–12).

Thus, an obvious direct linkage between production and consumption of pulses was not seen at district level, and it becomes important to identify other factors associated with pulse consumption.

### 3.2 Factors associated with pulse consumption

A summary of household characteristics is provided in Supplemental Table 3. The average household size was four, average household monthly expenditure was Rs 7211 (USD136.5) where pulses accounted for 6.5% of the total food expenditure. The average years of education for women in these households was about five years. Roughly 9% of households reported consuming pulses from own production.

Districts were located on an average 75.7 km away from a tier I or II city and the average price of pulses was Rs. 55/kg (~USD 1.04). The average price of other food items (excluding pulses) was Rs. 28/kg (~USD 0.34). The variance partition coefficient (VPC) for null model was 23.18% and this was the percentage of the total variance of households’ intake of pulses in the null model that could be attributed to district factors. The VPC is large enough to consider district level variability and district level covariates that could account for that variability through multi-level model with household as level 1 and district as level 2 (households nested within districts).

#### 3.2.1 Household characteristics

Household-level characteristics, such as number of members, total land owned (in hectares), maximum educational attainment by a woman in the household, whether the household belonged to General category or had a PDS card, were positively and significantly associated with household annual pulse intake (all p < 0.05, Table 3).

**Table 3:** Regression coefficient of household and district characteristics in multilevel model for household pulse consumption in NSSO data 2011-12.

Dependent Variable: Household annual consumption of pulses (kg)	Regression Coefficient	Robust Std. Err.	[95% Conf. Interval]	
Household Monthly Consumption Expenditure (Rs per month)	0.00188	0.00007	0.00174	0.00201
Household size	5.12364	0.11915	4.89011	5.35716
Total land owned (Hectares)	0.54683	0.12775	0.29644	0.79721
Maximum Educational Attainment by a woman in the household (years)	0.09449	0.03918	0.01770	0.17128
General Category (1- belongs to general category, 0 – SC/ST/Others)	1.05055	0.31352	0.43607	1.66503
PDS beneficiary (1 - HH is a PDS beneficiary, 0 - otherwise)	2.41435	0.31466	1.79763	3.03107
HH's consumption from own production (1 - if household consumed pulses from own production, 0 - otherwise)	5.03725	0.55707	3.94542	6.12908
District Production Surplus of Pulses (adjusted for production loss) (kg)	2.73 × 10^−8^	1.13 × 10^−8^	5.19 × 10^−9^	4.94 × 10^−8^
Distance of district to nearest city (km)	−0.00769	0.00287	−0.01331	−0.00207
Median monthly per capita expenditure of district (Rs per month)	0.00383	0.00116	0.00156	0.00610
Median price of pulses in district (Rs)	−0.10129	0.05060	−0.20047	−0.00212
District Price of All Foods excluding pulses (Rs/kg)	−0.39925	0.08506	−0.56596	−0.23254
constant	15.02687	2.72873	9.67867	20.37508

#### 3.2.2 Pulse availability

Households that reported pulse intake from own production had higher intakes (median annual household intake of 52.4 kg V 36.5 kg, p < 0.01 using Mann-Whitney-U test), although median per capita intakes did not differ greatly between the two categories (median annual per capita intake of 9.9 kg V 9.3 kg, p < 0.01 using Mann-Whitney-U test). About 96% of households that consumed pulses from own production lived in rural areas. The MLM results indicate that, a household that consumes from own production has an annual intake greater by 5 kg (95%CI: [3.95, 6.13]) (Table 3).

District-level local availability of pulses measured as production surplus matters; the median per capita annual pulse intake of high-producing districts (10 kg; n = 167) was higher than that of low-producing districts (8.7 kg) (p < 0.01, using Mann Whitney *U* test), based on a cut-off of national per capita production of 9.51kg/annum. The MLM results indicate that an increase in the annual district production surplus of pulses by 0.05 million tonnes could improve the annual intake of pulses of households located in that district by 1.865 kg (95% CI:[0.26, 2.47]) on average (Table 3).

#### 3.2.3 Pulse accessibility

Accessibility to markets is important in determining a household's intake of pulses, as a 100 km increase in the distance of the district to the nearest city can lower household annual intake of pulses by 0.77 kg (95%CI:[-1.33, -0.21]) on average (Table 3).

#### 3.2.4 Pulse affordability

Income and price affordability are important determinants of household pulse intake, with higher household income (monthly expenditure taken as proxy variable) and lower price of pulses in the district facilitating higher household pulse intakes, after controlling for price of other food items in the district (p < 0.01 for household monthly expenditure and district price of other food items, p = 0.045 for district price of pulses) (Table 3). There was an interaction between own production of pulses and wealth such that higher consumption among households that reported pulse intake from own production increased with wealth. The regression coefficients were higher for the upper wealth quintiles (regression coefficient = 16.45, 95% CI: 12.92, 19.99) compared to the reference lowest quintile (Supplemental Figure 2).

When we additionally explored the role of religion (1- Jainism, Hinduism; 0 – otherwise) in determining household pulse consumption, the statistical significance of availability, accessibility and price affordability changed, though the direction and the effects sizes did not change (Supplemental Table 4). Household pulse intake was higher for Hindu and Jain households (p < 0.01) (Supplemental Table 4). District pulse availability measured as production surplus, market accessibility and pulse affordability were also significantly associated with pulse intakes by males in the household (Supplemental Table 5).

## 4 Discussion

There is a large body of evidence exploring the role of affordability and availability, albeit separately, in predicting food demand for the future and estimating the capacity to provide the same through supply projections, using demand-system and production-system models (Kumar et al., 2010, 2011, 2016, 2017; Minocha et al., 2019). But these studies were limited in exploring the role of market accessibility in determining household food demand. The present study attempted to analyse the role of market accessibility, affordability and production availability of pulses within a single framework using nationally representative survey datasets. The findings contribute to the growing literature to aid policy formulation in making protein-rich foods available and accessible at an affordable price.

At the national level, pulse consumption outweighed production (Table 1) and was therefore supplemented by imports. In 2018–19 the total pulse production for India stood at 23.22 million tonnes (Area and Production Statistics, 2019), which was supplemented by imports of 2.34 million tonnes whereas exports amounted to 0.24 million tonnes (India's Position as an Importing Country for Pulses), and India's reliance on pulse imports to meet the demand continues to grow. When availability at a district level as local pulse production surplus was considered, there was a high variation in pulse production and therefore local availability across the Indian districts, with 60 districts producing the quantity sufficient to meet the requirement. But these did not translate to higher variation in pulse consumption, and the consumption was well below the recommended intake (Fig. 2, Fig. 3, National Institute of Nutrition, 2011) even in the 60 districts producing sufficient quantity.

Although the intake of pulses was sub-optimal, household pulse intake was positively associated with district availability of pulses when examined using the multilevel model. Household level pulse production was also associated with pulse consumption as shown in other studies (Bundala et al., 2020; Mfikwa and Kilima, 2014) and most of these households were located in rural areas. Several studies also suggest that higher production and farm diversity is associated with greater diet diversity, especially among children in farming households (Demeke et al., 2017; Hirvonen and Hoddinott, 2014; Jones et al., 2014; Mulmi et al., 2017).

About 66% of India's population resides in rural areas, depending on agriculture as a source of income as well as to meet their food security requirements (Rural population as % of population) and advocating pulse production to agricultural households has the potential to improve quality plant protein intake and to create income opportunities (Aberman and Roopnaraine, 2018). However, the decision to produce to improve intakes are based on factors such as quantities to be retained or sold and these decisions are dependent on food security needs, financial needs, quantity produced, volatility in pricing, among others (Aberman and Roopnaraine, 2018; Ferdous et al., 2016; Talukder et al., 2010; Tesfaye and Tirivayi, 2020). Factors external to the household, such as supply factors of scale of production, reducing the gap between farm harvest and market prices, improving availability of certified quality seeds, providing extension services and technical knowledge, introduction of pulse processing units, among others can in turn bolster rural household pulse production (Pandey et al., 2019; Reddy and Reddy, 2010; Smith et al., 2018) and potentially consumption.

Rethinking the policy on MSP, to either raise it and engage in governmental pulse procurement to raise farm-gate prices, or to do away with it entirely, so that farmers are offered market-competitive prices, could help address supply issues (Joshi et al., 2016). But pulse production might benefit wealthier farming households more than their poorer counterparts, as the association of household production and consumption was stronger in higher wealth quintiles (Supplemental Figure 2). This could reflect larger farm sizes owned by wealthier farming households, and higher pulse output by such households. Factors such as higher crop diversity, extent of commercialization and socio-economic factors such as land-holding size and educational attainment have been shown to positively influence dietary diversity of wealthier farming households (Singh et al., 2020).

This study showed that accessibility to markets is as equally important as affordability and availability. Better market accessibility, as deduced by reduced market distance or reduced walking time, have been found to have the same effect on dietary diversity as producing an additional food group (Koppmair et al., 2016; Sibhatu et al., 2015). Better market accessibility also weakens the association between production and diet diversity, as observed for villages located closer to markets (Hirvonen and Hoddinott, 2014; Luckett et al., 2015).

Our results indicate a significant and positive association of distance to the nearest city (as an indicator of market) with household pulse intake. Since the average distance to the market (in our case, the nearest city) was 75 km, this suggests the existence of small towns closer to the villages which act as a bridge between cities and villages. Not only do these small towns act as markets for agriculture produce from these rural regions and improve agriculture capabilities in these regions by providing agriculture extension services, but they are also centres of distribution of goods, including food and services to rural populations (Tacoli and Agergaard, 2017). Nearby urbanization could lead to better rural nutrition through improved access to a wider range of goods including nutrient-dense foods such as fruits, vegetables and animal products (Gómez and Ricketts, 2013).

The present study contributes to the evidence that improving market accessibility by setting up new markets or improving existing market channels can improve pulse intake. Due to the existence of both formal and informal market channels in many developing economies, the role of market accessibility in food demand is complex and rarely studied (FAO, 2003; Vorley, 2013). In India, value chains differ across districts and involve multiple stakeholders, even for a single food commodity (Mishra and Dey, 2018), hence the complexity in understanding the system increases. Given this, more research is required to understand how accessibility can be improved to improve quality food intake.

Affordability also influences household consumption; household pulse intake was positively associated with household monthly expenditure (as a proxy for household income) and negatively associated with district price of pulses, after controlling for prices of other foods. Pulse consumption was found to increase across income categories and were relatively income-inelastic (i.e., |elasticity| < 1) which indicates that with a rise in income, quantity demanded of pulses will increase, but less than proportionately (Kumar et al., 2011). Pulses were also found to be relatively price-inelastic (i.e., |elasticity| < 1) and in the event of price inflation, pulse intake was likely to decline (Kumar et al., 2011). Lower income groups were found to be more sensitive to price and income change (Kumar et al., 2011, 2017). While imports supplement pulse availability and in theory could lower price of pulses, currently they have a moderating effect on pulse prices (Negi and Roy, 2015).

India's population meets between 49% and 58% of its protein requirements through cereal based diets (National Sample Survey Office, 2014b), which continue to be available at highly subsidized rates through mechanisms such as the Public Distribution System (NITI Aayog, 2016). We observe a positive association between household pulse consumption from market purchase and own production, and PDS beneficiary status (Table 3), which could be attributed to the allocation of money saved on cereals to other food items such as pulses, edible oil, vegetables and sugar (Kishore and Chakrabarti, 2015; Shrinivas et al., 2018). Currently, the evidence of impact of increased cereal subsidy and improved targeting on pulse consumption and subsequent nutrition is mixed, as available literature differ on methodologies adopted and regions studied for evaluating the impact (Kaushal and Muchomba, 2015; Kishore and Chakrabarti, 2015; Rahman, 2016; Shrinivas et al., 2018). While many states have taken up the disbursal of pulses through PDS at subsidized rates, the impact of this on total pulse consumption may remain quite small (Chakrabarti et al., 2016). A deeper analysis of demand-side factors to improve pulse consumption is required.

There are certain limitations to this study. As the National Sample Survey Office did not release any large-sample data on consumption after CES 2011–12, the study was limited to analysing pulse consumption and production in India for 2011–12. However, even with this data being the most recent available of its kind, this analysis is still pertinent to understanding the dynamics of consumption even if the information is a decade old. As demand and supply system models were not used to explore pulse affordability and availability respectively, care must be taken in the interpretation of the multilevel modelling results.

A similar approach in assessing affordability has also been adopted in other studies by controlling for price of other food items (Choudhury et al., 2020). The model was also limited in assessing market accessibility as distance of the district to the nearest city, as NSSO CES did not collect data on markets accessed by households. Though data on market and accessibility for consumption are available for a few districts in the Village Dynamics in South Asia (VDSA) study, this data could not be used due to its limited coverage. The study compared district pulse consumption with district aggregate recommended intake based on the Indian National Institute of Nutrition's recommended per capita pulse intake of 80 g/day (National Institute of Nutrition, 2011), but this recommendation is for a moderately active person. In reality, the recommended intake varies based on age, gender, type of diet and level of physical activity.

To summarize, this study provides evidence of linkages between district-level production and household consumption of pulses in India. But the pattern of production and consumption leaves much to be desired, with evidence indicating insufficient intakes even among households in high-producing districts. While much of the focus of existing studies has been on improving production and productivity of pulses, the findings from this study indicate that there are many interacting factors covering availability, affordability and accessibility which determine a household's choice of consumption altogether. Policies that are targeted towards improving affordability of pulses, household production of pulses especially for rural households and market accessibility would have more merit. But for this, a deeper analysis at the local context is required.

## Funding

This work was supported by the 10.13039/100000865Bill & Melinda Gates Foundation, Seattle, WA [Grant Number: OPP1194964].

## Role of funding organization

The Bill & Melinda Gates Foundation did not have any role in the study design, analysis, interpretation of data, writing of the report or the decision to submit the article for publication.

## Declaration of competing interest

The authors declare that they have no known competing financial interests or personal relationships that could have appeared to influence the work reported in this paper.

## Figures and Tables

**figs1:**