# Supplementary material for: Factors influencing household pulse consumption in India: A multilevel model analysis
Source: Glob Food Sec. 2021 Jun;29:100534. doi: 10.1016/j.gfs.2021.100534 (PMC8202232; doi:10.1016/j.gfs.2021.100534)
Supplement: Multimedia component 2 [file mmc2.docx]

| **Pulse type** | **Share in production (%)*** | **Share in consumption (%)*** |
| --- | --- | --- |
| Pigeon Pea | 16.5 | 30.9 |
| Chickpea | 47.1 | 23.8 |
| Green Gram | 11.1 | 12.9 |
| Red Lentils | 6.7 | 13.9 |
| Black Gram | 11.5 | 11.3 |
| Dried Peas | 4.7 | 5.9 |
| Grass Pea | 2.5 | 1.4 |
