# Supplementary material for: Factors influencing household pulse consumption in India: A multilevel model analysis
Source: Glob Food Sec. 2021 Jun;29:100534. doi: 10.1016/j.gfs.2021.100534 (PMC8202232; doi:10.1016/j.gfs.2021.100534)
Supplement: Multimedia component 3 [file mmc3.docx]

| **Production-Consumption Matrix** | | **Production compared to median for India** | |
| --- | --- | --- | --- |
|  |  | High | Low |
| **Consumption compared to median for India** | High | 78 (12.5%) | 138 (22.2%) |
|  | Low | 89 (14.3%) | 320 (51.2%) |
