# Supplementary material for: Factors influencing household pulse consumption in India: A multilevel model analysis
Source: Glob Food Sec. 2021 Jun;29:100534. doi: 10.1016/j.gfs.2021.100534 (PMC8202232; doi:10.1016/j.gfs.2021.100534)
Supplement: Multimedia component 4 [file mmc4.docx]

| **Variable** | **Mean** | **Std. Dev.** | **Min** | **Max** |
| --- | --- | --- | --- | --- |
| Dependent Variable: Household annual consumption of pulses (kg) | 42.33 | 29.16 | 0.00 | 975.00 |
| Household Monthly Consumption Expenditure (Rs per month) | 7211.30 | 6635.61 | 44.11 | 339831.70 |
| Household size | 4.43 | 2.21 | 1.00 | 39.00 |
| Total land owned (Hectares) | 0.50 | 1.33 | 0.00 | 120.67 |
| Maximum Educational Attainment by a woman in the household (years) | 5.35 | 3.87 | 0.00 | 13.00 |
| General Category (1- belongs to general category, 0 – SC/ST/Others) | 0.29 | 0.45 | 0.00 | 1.00 |
| Religion (1- Hindu and Jainism; 0 - otherwise) | 0.83 | 0.37 | 0 | 1 |
| PDS beneficiary (1 - HH is a PDS beneficiary, 0 - otherwise) | 0.80 | 0.40 | 0.00 | 1.00 |
| HH's consumption from own production (1 - if household consumed pulses from own production, 0 - otherwise) | 0.09 | 0.29 | 0.00 | 1.00 |
| District Production Surplus of Pulses (adjusted for production loss) (kg) | -1.2 x10^7^ | 4.04 x10^7^ | -12.9 x10^7^ | 19.1 x10^7^ |
| Distance of district to nearest city (km) | 75.67 | 61.18 | 0.16 | 1627.58 |
| Median Monthly per capita Expenditure of District (Rs per month) | 1494.02 | 616.90 | 560.26 | 3761.60 |
| Median price of pulses in district (Rs) | 55.41 | 7.16 | 30.57 | 86.52 |
| District Price of All Foods excluding pulses (Rs/kg) | 28.18 | 7.08 | 15.92 | 52.86 |
| No. of observations | 101055 | | | |
| No. of districts | 623 | | | |
