# Supplementary material for: Factors influencing household pulse consumption in India: A multilevel model analysis
Source: Glob Food Sec. 2021 Jun;29:100534. doi: 10.1016/j.gfs.2021.100534 (PMC8202232; doi:10.1016/j.gfs.2021.100534)
Supplement: Multimedia component 5 [file mmc5.docx]

| **Dependent Variable: Household annual consumption of pulses (kg)** | **Coef.** | **Robust Std. Err.** | **[95% Conf. Interval]** | |
| --- | --- | --- | --- | --- |
| Household Monthly Consumption Expenditure (Rs per month) | 0.00188 | 0.0000685 | 0.00174 | 0.00201 |
| Household size | 5.12855 | 0.11941 | 4.89451 | 5.36259 |
| Total land owned (Hectares) | 0.54093 | 0.12620 | 0.29358 | 0.78829 |
| Maximum Educational Attainment by a woman in the household (years) | 0.09163 | 0.03939 | 0.01443 | 0.16884 |
| General Category (1- belongs to general category, 0 – SC/ST/Others) | 1.01663 | 0.30949 | 0.41004 | 1.62323 |
| Religion (1- Hindu and Jainism; 0 - otherwise) | 0.84905 | 0.32144 | 0.21903 | 1.47907 |
| PDS beneficiary (1 - HH is a PDS beneficiary, 0 - otherwise) | 2.40004 | 0.31201 | 1.78850 | 3.01157 |
| HH's consumption from own production (1 - if household consumed pulses from own production, 0 - otherwise) | 5.01562 | 0.55466 | 3.92850 | 6.10273 |
| District Production Surplus of Pulses (adjusted for production loss) (kg) | 2.43 x 10^-8^ | 1.17 x 10^-8^ | 1.28 x 10^-9^ | 4.73 x 10^-8^ |
| Distance of district to nearest city (km) | -0.00606 | 0.00316 | -0.01225 | 0.00013 |
| Median Monthly per capita Expenditure of District (Rs per month) | 0.00367 | 0.00119 | 0.00133 | 0.00601 |
| Median price of pulses in district (Rs) | -0.09506 | 0.05339 | -0.19971 | 0.00959 |
| District Price of All Foods excluding pulses (Rs/kg) | -0.41552 | 0.08666 | -0.58536 | -0.24567 |
| constant | 14.56210 | 2.94181 | 8.79626 | 20.32794 |
