# Supplementary material for: Factors influencing household pulse consumption in India: A multilevel model analysis
Source: Glob Food Sec. 2021 Jun;29:100534. doi: 10.1016/j.gfs.2021.100534 (PMC8202232; doi:10.1016/j.gfs.2021.100534)
Supplement: Multimedia component 6 [file mmc6.docx]

| Dependent Variable: Annual consumption of pulses by adult males in the HH (kg) | Coef. | Robust Std. Err. | [95% Conf. Interval] | |
| --- | --- | --- | --- | --- |
| Household Monthly Consumption Expenditure (Rs per month) | 0.0010 | 0.0000 | 0.0010 | 0.0011 |
| Household size | 1.0932 | 0.0555 | 0.9845 | 1.2020 |
| Total land owned (Hectares) | 0.6944 | 0.0719 | 0.5535 | 0.8353 |
| Maximum Educational Attainment by a woman in the household (years) | -0.1532 | 0.0244 | -0.2011 | -0.1054 |
| General Category (1- belongs to general category, 0 – SC/ST/Others) | 1.1489 | 0.1915 | 0.7735 | 1.5244 |
| PDS beneficiary (1 - HH is a PDS beneficiary, 0 - otherwise) | 1.6835 | 0.1728 | 1.3448 | 2.0222 |
| HH's consumption from own production (1 - if household consumed pulses from own production, 0 - otherwise) | 1.9604 | 0.3129 | 1.3472 | 2.5736 |
| District Production Surplus of Pulses (adjusted for production loss) (kg) | 1.72 X 10^-8^ | 7.87 X 10^-9^ | 1.80 X 10^-9^ | 3.27 X 10^-8^ |
| Distance of district to nearest city (km) | -0.0039 | 0.0015 | -0.0070 | -0.0009 |
| Median Monthly per capita Expenditure of District (Rs per month) | 0.0044 | 0.0008 | 0.0029 | 0.0059 |
| Median price of pulses in district (Rs) | -0.1701 | 0.0362 | -0.2410 | -0.0993 |
| District Price of All Foods excluding pulses (Rs/kg) | -0.2308 | 0.0564 | -0.3412 | -0.1203 |
| constant | 15.3531 | 1.8925 | 11.6438 | 19.0623 |
