# Supplementary material for: Factors influencing household pulse consumption in India: A multilevel model analysis
Source: Glob Food Sec. 2021 Jun;29:100534. doi: 10.1016/j.gfs.2021.100534 (PMC8202232; doi:10.1016/j.gfs.2021.100534)
Supplement: Supplementary file 6 [file mmc1.docx]

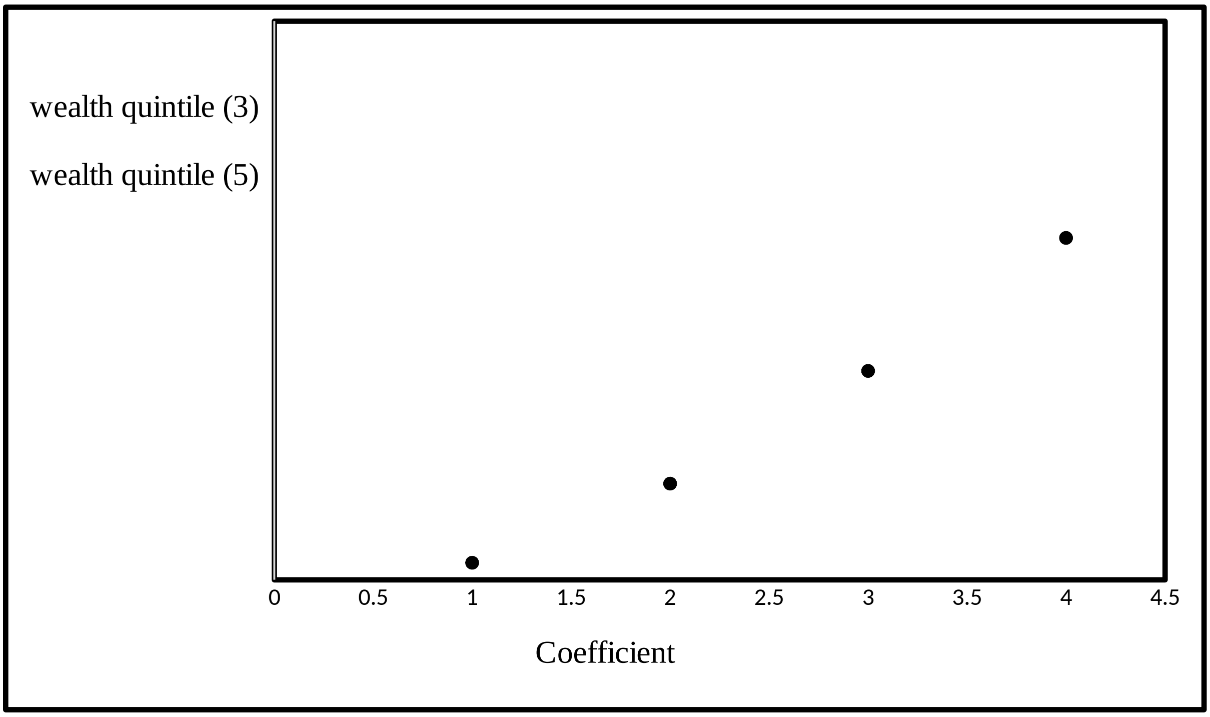
wealth quintile (3)
wealth quintile (5)
0
0.5
1
1.5
2
2.5
3
3.5
4
4.5
Coefficient
